# A cognitive forcing tool to mitigate cognitive bias – a randomised control trial

**DOI:** 10.1186/s12909-018-1444-3

**Published:** 2019-01-08

**Authors:** Eoin D. O’Sullivan, Susie J. Schofield

**Affiliations:** 1 Department of Renal Medicine, Royal Infirmary of Edinburgh, 51 Little France Cres, Edinburgh, EH16 4SA UK; 2 Centre for Medical Education, University of Dundee, Dundee, UK

**Keywords:** Cognitive bias, Heuristics, Clinical error, Decision making

## Abstract

**Background:**

Cognitive bias is an important source of diagnostic error yet is a challenging area to understand and teach. Our aim was to determine whether a cognitive forcing tool can reduce the rates of error in clinical decision making. A secondary objective was to understand the process by which this effect might occur.

**Methods:**

We hypothesised that using a cognitive forcing tool would reduce diagnostic error rates. To test this hypothesis, a novel online case-based approach was used to conduct a single blinded randomized clinical trial conducted from January 2017 to September 2018. In addition, a qualitative series of “think aloud” interviews were conducted with 20 doctors from a UK teaching hospital in 2018. The primary outcome was the diagnostic error rate when solving bias inducing clinical vignettes. A volunteer sample of medical professionals from across the UK, Republic of Ireland and North America. They ranged in seniority from medical student to Attending Physician.

**Results:**

Seventy six participants were included in the study. The data showed doctors of all grades routinely made errors related to cognitive bias. There was no difference in error rates between groups (mean 2.8 cases correct in intervention vs 3.1 in control group, 95% CI -0.94 – 0.45 *P* = 0.49). The qualitative protocol revealed that the cognitive forcing strategy was well received and a produced a subjectively positive impact on doctors’ accuracy and thoughtfulness in clinical cases.

**Conclusions:**

The quantitative data failed to show an improvement in accuracy despite a positive qualitative experience. There is insufficient evidence to recommend this tool in clinical practice, however the qualitative data suggests such an approach has some merit and face validity to users.

**Electronic supplementary material:**

The online version of this article (10.1186/s12909-018-1444-3) contains supplementary material, which is available to authorized users.

## Background

The term “cognitive bias” describes a variety of unconscious influences, short-cuts and behaviours which influence our decision making. Cognitive bias is increasingly recognised as an important cause of medical error. Cognitive factors are estimated to contribute to up to 75% of errors in internal medicine and errors in cognition have been identified in all steps of the diagnostic process including information gathering, association triggering, context formulation, processing, and verification [1, 2]. Supporting this, a retrospective cohort analysis of errors at a veteran’s affairs facility suggested at least 13% of diagnostic errors relate to interpretation of test results and 78.9% involve the patient encounter [3]. Indeed, when doctors are asked to reflect on their own errors, cognitive error is thought to be responsible for up to 30% of self-reported diagnostic errors in the emergency department and 42% in internal medicine [4, 5].

Despite this growing recognition of cognitive error, it has proven a challenging area to research for a variety of reasons, including a lack of high quality data on prevalence, a lack of granularity when data are present and difficulty recording or observing a sometimes nebulous and intangible internal process [6–9]. Graber et al. have complied a review of potential interventions designed to reduce overall diagnostic error, a subgroup of which includes cognitive bias [10]. A recent review concludes that the majority of debiasing interventions are at least partially successful, although only 13 studies were targeted at health care professionals, and the methodological quality was highly variable [11]. Debiasing is a rapidly growing area of research and over 40 potential mitigation strategies have been identified and explored in recent publications. Understanding how and when to deploy a debiasing strategy, and measuring its effect remains challenging [12]. This is the important gap in the evidence we wished to address with our research. Our aim was to design and test a debiasing strategy in clinicians.

Characteristics of an ideal debiasing strategy are efficacy, ease of deployment, and specificity to the task at hand. After reviewing the literature for previous efforts to debias clinicians, and considering our desired characteristics, we decided a simple mnemonic would be an ideal debiasing tool. To assess our tool, we conducted a randomised controlled trial of its use during clinical cases by clinicians. To improve our understanding of the tool, an additional qualitative approach using a think-aloud protocol was used.

While lists have been generated of likely biases within medicine, it is unknown as to which biases are the most statistically common, the most clinically impactful and would thus result in the highest yield if targeted by a successful intervention [13]. Previous research has not been directed at the most common biases, but usually at a convenience sample of easily investigated biases. We thought it important to design our cognitive intervention and cases specifically towards the most common biases reported in the literature, to maximise any potential value such an intervention would have. Those biases that have been reported experimentally or observationally in the literature are described in Additional file 1, and these informed both our forcing strategy and clinical cases. While many other biases undoubtedly exist, we felt designing our cases and interventions in such a way was most in keeping with the philosophy of evidence-based medicine.

## Methods

**Fig. 1:**
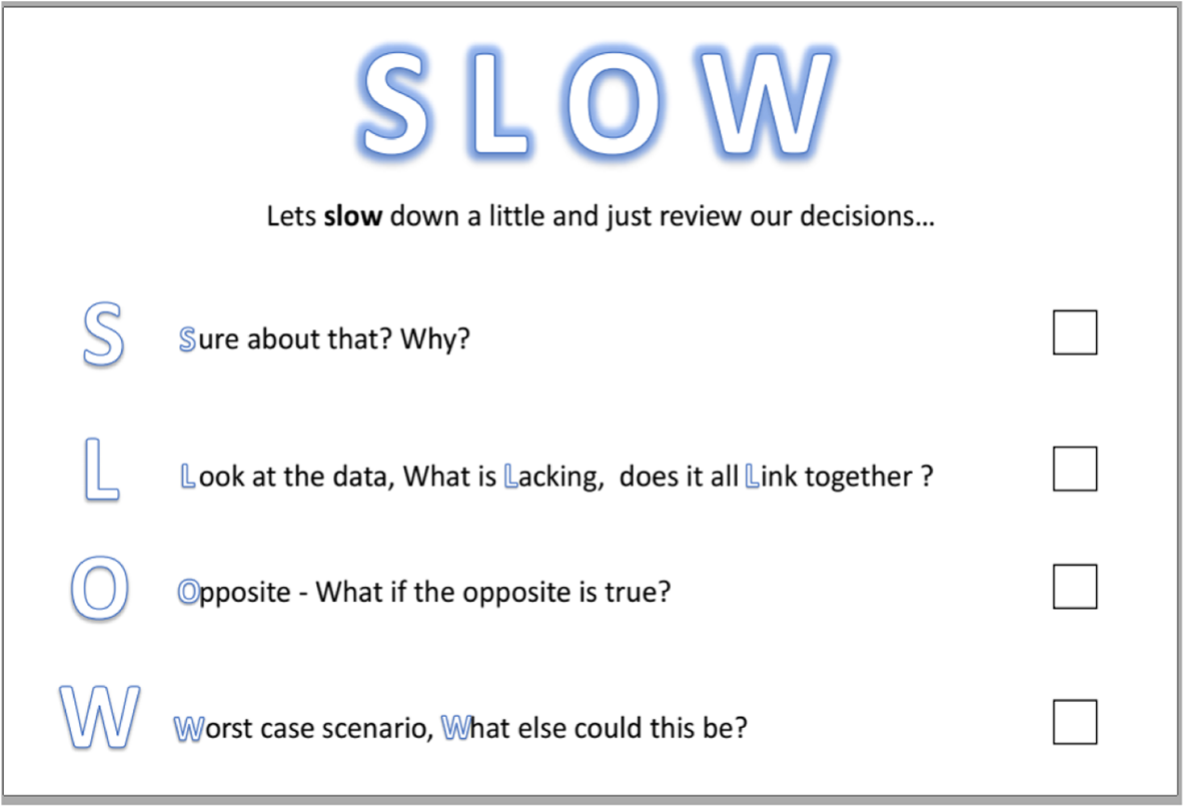
The “SLOW” cognitive forcing tool as displayed to participants

The mnemonic “SLOW” was created as our intervention. The word “slow” itself is an important reminder to slow down, an evidenced based method of improving diagnostic accuracy [14–18]. In addition to this, each letter is a prompt, which is chosen to counteract a specific bias. Each specific prompt is designed to act as a metacognitive trigger, drawing from the existing evidence of individually successful interventions and combining these into a single tool. It is possible a single metacognitive trigger may have an effect on multiple forms of bias. The “SLOW” intervention presented to students and doctors is shown in Fig. 1. Table 1 summarises the specific biases that have been shown to improve with each trigger used, as well as the evidence for this.

**Table 1:** The biases addressed by the SLOW tool

Intervention	Bias addressed	Reference
Slowing Down	Availability, multiple	[14–18, 24–26]
S – Sure?	Overconfidence, hindsight	[27–29]
L – Look /Lacking/Link	Multiple	[30]
O – Opposite	Anchoring	[31–33]
W – Worst case Scenario	Search satisfying	[30]

### Qualitative methods

In order to create some understanding of how the SLOW tool might have an effect, an additional qualitative approach was employed. Subjects recruited to the qualitative study were an additional convenience sample of students and doctors within the Royal Infirmary of Edinburgh, UK.

A thematic analysis of the qualitative data was performed. Volunteers were given the case scenarios to consider and asked to answer questions that followed. The first 25% of volunteers were used as a control group to establish common themes and define some of the cognitive missteps that occur as they moved towards the bias. The following 75% were then introduced to the cognitive forcing tool and asked to use the tool when answering the questions.

Data were gathered using a concurrent think-aloud protocol, the interviews were recorded and stored digitally. The think aloud protocols were then transcribed verbatim. After data reduction, open-coding was performed on all transcripts where thought processes, key concepts and biases were coded and quantitatively analysed.

Ten cases were designed to trigger a specific bias. Each case is a short clinical scenario with subsequent questions. To hide the potential biases from candidates and emulate the real-world nature of diagnostic error, attention diverting information and distractors were used when necessary. Two pilot studies were performed to ensure the cases triggered bias and to ensure clarity and content validity. The biases covered include the representative bias, conjunction fallacy, overconfidence, base rate neglect, diagnostic momentum, ascertainment bias, the framing effect, conjunction rule and availability bias. The authors were satisfied that participants in these pilot studies understood the questions being asked and were considering the questions as intended. Full cases can be found in Additional file 2.

### Quantitative methods

The SLOW intervention was tested as a randomised control trial delivered via an online application. A domain was registered, and the cases (Additional file 2) were presented to candidates who followed a shared hyperlink. After reviewing a consent form, each volunteer was randomised by the application to either an intervention or control group in a 50:50 ratio using a random number generator at the time of participation. To be eligible to participate, volunteers were required to be medical professionals of any seniority or discipline, or medical students. The active group was given a brief primer on the SLOW mnemonic and how to use it. Following each case, the subjects were subsequently asked to complete an onscreen checklist prompted by the “SLOW” tool. This checklist was implemented to force the clinician to consider each specific cognitive element before they could proceed to the following question. A timer was visible on screen counting upwards to induce a sense of urgency and time pressure, although no time limit was placed on the cases. The second group acted as a control group and solved the same cases but without the SLOW prompt.

These two groups were then compared under the hypothesis that the SLOW forcing tool would increase the number of correct answers in the intervention arm.

### Recruitment

Subjects recruited included senior medical students, junior doctors and consultants. Participants were recruited locally in Edinburgh via the medical school and local mailing lists. University College Cork, Ireland and University of Dundee, UK assisted in recruitment of further undergraduates. Social media was also used as a recruitment tool to recruit post graduate doctors, including posting on medical Facebook groups, instant messaging groups, and Twitter.

### Outcomes

The primary outcomes were the number of correct answers in the intervention group as compared to control group.

Secondary outcomes include comparing the effect of any clinical seniority, age or time taken on error rates. We also examined the error rates of each question individually to assess any effect of the intervention on specific biases or scenarios.

### Statistical analysis

Calculating a sample size when the estimated effect size is unknown is challenging. Based on our pilot studies we anticipated a mean correct response of 50% + − 10%. We estimated that the SLOW tool would deliver an increase of 10%. Thus, a statistical effect size of this magnitude required approximately 168 students in total to have a 90% chance of detecting an effect as significant at the 5% level.

The free text answers to questions were coded into nominal (binary) variables for analysis. Continuous variables are expressed as means and standard deviations. Chi-squared and independent students t-tests were used for comparison of nominal and continuous data respectively.

All statistics were performed in SPSS version 20. All *p* values are two-sided at a 0.05 significance level. During the analysis of the trial results, the investigators were blinded to the group type during analysis.

### Ethics and data management

Ethical approval was gained via University of Dundee Research Ethics committee. All data were stored anonymously under 256-bit AES encryption on a single solid-state hard drive in a secure location. The British Educational Research Association ethical guidelines were considered during study design and adhered to through this research [19].

## Results

### Qualitative results

The aim of the qualitative analysis was to try to understand common trends that lead to incorrect answers. This could potentially inform us as to “why” people were making cognitive mistakes. Our qualitative analysis included even numbers of men (*n* = 10; 50%) and women (n = 10; 50%), and included 5 students, 3 first year graduates, and the remainder were varying degrees of seniority between 2 and 10 years post-graduation. Data were gathered using the “think aloud” protocol, and were then assessed for quality [20]. Each interview was assessed to ensure it was of high enough quality to interpret. Once common themes became apparent for each case, further cases were used to illuminate differences in findings, and once the same themes were emerging, it was felt that saturation of the data had occurred which added face validity to the findings.

The most striking aspect of the data is the prevalence of error. Even experienced clinicians were prone to the same errors students made. This suggests to the authors that bias may only be partially compensated for by clinical experience. Subjects rarely changed their minds once they had answered a question – as change of mind was slightly more common when using the SLOW tool and they were forced to reconsider data. There was a tendency to immediately frame the problem as soon as a pattern was recognised (e.g. “This is a situation of drug seeking behaviour” in the case of hidden infection or “this is a question about statistics isn’t it?” during a case demonstrating drug marketing techniques). This initial framing informed how the remaining information was processed. When this initial framing was incorrect, it invariably lead to error. Using the SLOW tool helped many candidates return to the beginning and purposely reframe the question, which often lead to improvements. “Considering the Opposite” was also a powerful technique and many subjects who had provided incorrect answers or made errors, reconsidered and “safety netted” following the SLOW tool. While this didn’t necessarily increase their initial diagnostic accuracy, the clinical plans were safer and less likely to result in downstream error.

Detailed case by case analysis is provided in Additional file 3.

### Quantitative results

We recruited 300 medical professionals from a number of centres from across the UK, Republic of Ireland and North America (locations not recorded to maintain anonymity but noted through feedback to the investigators) from January 2017 to September 2018. Following data cleaning 244 responses were felt to be insufficient or incomplete, leaving 76 participants of sufficient quality for analysis. Most of excluded cases answered 1 or 2 questions initially before quitting the application and these have not been included in further analysis. This gives a “drop-out” rate of 74.6%. Table 2 shows the characteristics of our candidates.

**Table 2:** Characteristics of candidates

	Intervention(*n* = 37)	Control(n = 37)
Level of Training		
Student	0	2
Junior	11	13
Middle Grade	24	17
Senior	2	5
Age (years)		
18–25	11	12
26–34	21	20
35+	5	5

### Primary outcome

The primary outcome the number of correct responses to the cases. The intervention group had a mean of 2.8 correct answers compared to a mean of 3.1 correct answers in the control group. This difference was not clinically or statistically significant (*P* = .49, 95% CI -0.94 – 0.45).

### Secondary outcomes

#### Overall error rate of cases

The overall error rates are shown in Table 3. Error rate is the number of respondents who incorrectly answered the question. Our subjective impression based on the qualitative data that a large percentage of these errors are driven by the cognitive bias embedded into the question as designed. Qualitative interviews suggest that case 8 is probably not adequate to detect conjunction bias, but it is included for completeness. No difference was detected in error rates based on clinical grade or age range.

**Table 3:** Overall Error rates for each case

Case	Bias	Overall Error Rate
1	Representativeness	39 (52.7%)
2	Overconfidence	51 (68.9%)
3	Base Rate Neglect	73 (98.6%)
4	Confirmation Bias	42 (56.8%)
5	Search Satisfying	51 (68.9%)
6	Diagnostic Momentum	67 (90.5%)
7	The Framing Effect	56 (75.7%)
8	Conjunction Bias	66 (89.2%)
9	Framing Effect, Diagnostic Momentum	48 (64.9%)
10	Availability Bias	24 (32.4%)

#### Impact of intervention on individual cases

The “correct answer” response rates are summarised in Table 4. No difference was detected between the intervention and control group.

**Table 4:** Correct Response to Cases

	Intervention	Control	
	Frequency (%)	Frequency (%)	Sig.
Case 1	19 (51)	20 (54)	.816
Case 2	9 (24)	14 (38)	.209
Case 3	0	1 (3)	.555
Case 4	17 (46)	14 (38)	.434
Case 5	14 (38)	9 (24)	.209
Case 6	4 (11)	3 (8)	.691
Case 7	6 (16)	12 (33)	.104
Case 8	2 (5)	6 (16)	.134
Case 9	11 (20)	15 (41)	.330
Case 10	24 (65)	26 (70)	.619

#### Time taken

Time taken to complete each case was measured in seconds. Total time taken was 1472 ± 437 in the intervention group as compared to 1359 ± 403 in the control group (*P* = 0.37). Students-Test test failed to show any significant difference in the mean seconds taken to complete the individual cases between the intervention group and control group. No significant between group differences were found for any of the candidate characteristics.

## Discussion

Overall the results of our research are thought provoking. The findings of the qualitative think aloud protocols demonstrate that subjective quality of thought and problem solving improved with usage of the cognitive forcing tool.

The qualitative insight gained into the potential role of a debiasing tool is valuable. The participants feedback (Additional file 3) suggests the tool was successful in achieving its goal in helping users slow down and avoid bias. Furthermore, the SLOW intervention was more successful in some cases than others, being more useful in the case of “confirmation bias” but noticeably less useful in statistical bias for example. This is broadly encouraging and suggests there is a role for such a tool, but perhaps used in a more focused and refined manner.

These findings contrasted with the results of our trial which failed to show any difference in quality of answers by candidates using the intervention. There are a number of potential explanations for these discordant findings.

The most important explanation to consider is simply that the null hypothesis is true - that this intervention does not have any measurable effect on cognitive bias. The validity of such a statement however depends on the quality of the trial and analysis. Unfortunately, the recruited numbers were less than we hoped following data cleaning. An initial 300 responses dwindled to 78 when data were cleaned and prepared for analyses. The high attrition rate was due to incomplete answers, or clear attempts to rapidly get through each case as quickly as possible – as indicated by extremely short time spent per questions and single word/nonsense answers. Despite erring on the side of caution with a modest effect size in our initial power calculations, we were surprised by such a high rate of unusable data, and thus our recruitment may have been still too low to detect a difference between the groups.

A second potential explanation for the lack of difference between groups is limitations of the trial methodology. The cases themselves appear to have performed well and served their purpose and triggered a high number of incorrect answers as seen in Table 3. The qualitative conversations give some face validity to the notion that it was the specific biases leading to incorrect answers in each case, as per their design. However, it is interesting to note that there was no difference in the time taken between the intervention and the control group. This would suggest that ironically, despite the carefully considered name of the mnemonic, it failed to achieve at least one of its primary goals, which was to make candidates “slow down”. This leads us to wonder whether candidates engaged with the intervention as expected, or simply rapidly clicked through the options to progress to the next stage.

It may be unwise to attribute all incorrect responses to cognitive error, and some commentators argue that it is a lack of factual knowledge that is most important [21]. This has been vigorously disputed, and we would argue that our cases were designed so as not to need any advanced or complex knowledge to perform well [22].

We opted to use social media as a research platform to increase geographical reach and recruitment, and thus increased both generalisability of findings, and statistical power. Certainly, participants spanned all grades from student to consultant, and came from all over UK, the Republic of Ireland, and North America. However, we suspect that the anonymous, instantaneous and inpatient online culture lead to the very high dropout rate- dropout increased as subjects progressed through the cases. A further concern with using social media to recruit is confirming that participants were indeed doctors. While the channels used were specific to medical professionals, it would be impossible to confirm this without collecting identifiable information. While we are suggesting the trial data do not align entirely with the subjective assessments of our think aloud protocol, we are aware of the fact such qualitative data cannot provide objective evidence of efficacy. We are thus aware of the ironic risk of our own confirmation bias that might tempt us to “explain away” the trial shortcomings.

Limitations of the “think aloud” protocol are that in making inner speech external, there is an implicit simplification of abstract and complex thoughts, often before the speaker themselves can fully understand what they are about to say. It is unclear to psychological researchers, how much this vocalisation changes the thought itself as it is being verbalised. Ericson reasoned the goal of a think out loud protocol was to give access to the ‘working memory’ used when problem solving. This might pose potential issues however, such as only information that is noticed by the subject will be entered into working memory and thus be spoken. The limited capacity of working memory means that only words that immediately follow the thoughts represent what is currently happening in the working memory and taking too long to vocalise might result in newer thoughts replacing the original ones. These are thoughts which may never be captured if spontaneous speech is delayed. A second issue arises when considering think aloud protocols in the context of dual process thinking. System 1 is a non-verbal system, and probably the more bias prone system, as discussed. The act of verbalising a thought however, moves the problem to system 2. Given that system 2 is thought to have a greater capacity to recognise and overcome bias, this may obscure the true extent of bias in our subjects, and perhaps also explain the discrepancy between the benefits in the think aloud protocol as compared to the trial. Finally, there may be ‘intermediate’ or automatic processes, thoughts and connections which occur quickly in working memory which may be “unconscious” and might not be amenable to being captured by speech. Interpreting the thinking out loud data requires a degree of inference, based on interpretation of the subject’s tone, body language and nuances of the language (e.g. repetition of works when searching for meaning). This was an important additional necessity in data interpretation beyond simply reading through the text, as much meaning may be lost with a purely quantitative approach. Such an approach raises the issue of researcher subjectivity. Is was important to reflect upon the degree to which the first author was an “insider researcher” here, and the potentially confounding influence of this relationship [23].

The researcher in such a situation will always have some impact on a subject, so our pragmatic approach was to acknowledge, reflect on and attempt to mitigate this. We endeavoured to ignore any pre-existing knowledge of the subjects in ours mind, and continually challenged ourselves by questioning the impartiality of any assessments made. There may be some value in the position of an insider researcher during a think aloud protocol however – We felt that knowing some subjects for many years granted a very immediate and intimate understanding of their language choice and speech patterns and allowed us to identify their thoughts and subsequently their biases more readily. This was an advantage in some cases, and perhaps a think aloud protocol benefits from this insider phenomenon to an extent. Finally, one might also argue that in an era of pragmatic clinic trials, where researchers attempt to create as clinically relevant and realistic a trial as possible, failure to engage with an intervention in the comfort of their own homes /office may be a sign that doctors would not use such a tool on a busy ward.

Cognitive Biases can be deep seated and entrenched elements of our cognition, and it is likely that they exhibit heterogenous characteristics, having been established through a multitude of routes [12]. Thus, it is likely that a range of de-biasing strategies and regimes might be required- there is unlikely to a singular perfect solution. For example, our study was a single snapshot in time of bias post our intervention. It may however be important that such an intervention is repeated to improve or maintain its debiasing effect.

## Conclusion

Cognitive bias remains an important contributing factor to medical errors and at least some common bias can be reproduced in a written case-based format as we have done. When assessed using thinking aloud interviews, using a cognitive forcing strategy improved the subjective quality of thought and problem solving. These findings were not supported in a randomised controlled trial setting. Issues surrounding engagement with the intervention make it difficult to conclusively say whether this was a truly negative trial, or rather an unsuccessful trial design/delivery. We did not find sufficient evidence to support to use of this specific tool in routine clinical or education practice. Future researchers could learn from our experiences by ensuring that any interventions are deployed directly into clinical practice for a more pragmatic trial design which would allow them to bypass our concerns over engagement when not directly observed. What they would lose in potential subject number, they would gain in higher quality of data, and a directly observed intervention would allow them to assess real life engagement and clinical utility.

Cognitive bias remains an important area of future research, and we hope our results can perhaps guide others in designing their studies and tools to help the modern doctor navigate an increasingly complex world of clinical problems.

## Additional files

Additional file 1: Confirmed Bias in Clinical Medicine. A list of cognitive bias that has been specifically identified and reported within the literature. (DOCX 2182 kb)

Additional file 2: The clinical scenarios. Clinical vignettes designed to increase the chance of specific cognitive biases’ occurring in participants. (PDF 1458 kb)

Additional file 3: Qualitative interview summaries. More complete summaries of the themes emerging form the qualitative interviews. (DOCX 23 kb)

## Abbreviations

AES: Advanced Encryption Standard
UK: United Kingdom
