# Supplementary material for: A cognitive forcing tool to mitigate cognitive bias – a randomised control trial
Source: BMC Med Educ. 2019 Jan 8;19:12. doi: 10.1186/s12909-018-1444-3 (PMC6325867; doi:10.1186/s12909-018-1444-3)
Supplement: Supplementary file 2 — The clinical scenarios. Clinical vignettes designed to increase the chance of specific cognitive biases’ occurring in participants. (PDF 1458 kb) [file 12909_2018_1444_MOESM2_ESM.pdf]

Appendix 2

Case 1

It is late December and Gary attends your GP practice feeling unwell. He has had aches and pains in his joints and muscles for the last week, a sore throat, a headache and has been having chills and sweats at home.

Gary is health care worker who admits he has been "partying too hard" this last month. He tells you he has not been taking care of himself – he feels he has been drinking too much alcohol, doesn't get enough sleep and has skipped his routine occupational assessment and workplace vaccinations. He smokes 20 cigarettes per day and drinks 14 units of alcohol a week.

Gary tells you he has had unprotected sex with two men over the previous few months. This was a little out of character for Gary and he is clearly anxious about this.

You examine Gary and find he looks unwell, has a temperature of 38 °C (100.4 F), and some mild tender cervical lymphadenopathy. You weigh Gary and find that he has lost 4 kg in the month.

Q1. What feature concerns you most about Gary's case?

Q2. What is the most likely diagnosis?

Q3. What advice would you give Gary at this point?

00:34

[redacted]

Gary

Vital Signs

RR: 18 breaths/min

Temp: 38 °C

BP: 120/70 mmHg

HR: 90 bpm

O2 sats: 99% -room air

Next Case

Case 2

Ryan is a 28-year-old man, who presents to ED with worsening wheeze and dyspnoea. Ryan has had childhood asthma, but has not required any inhalers since he was a child. However, this last month, his wheeze has returned, and his GP had restarted his salbutamol inhalers and this has had some benefit until today, when Ryan's wheezing really became increasingly severe.
Ryan also tells you he has had some vague tingling in toes and feet recently, but thinks this started after straining his back while lifting heavy boxes.

Ryan has mild bilateral wheeze, and strikes you as looking very thin – he confirms that he has lost some weight recently, but he is not sure how much exactly. You find a confluent, macular rash with a small ulcer forming on his anterior shin – Ryan tells you he thinks he was kicked in this shin during a football game, but he isn't sure. It doesn't seem to be getting any worse. Ryan's urine dipstick is unremarkable

Test	Result	Range	Test	Result	Range
Hb (g/L)	157	135-180	Lymp ($\times 10^{9}$/L)	1.47	1.5-4
WCC ($\times 10^{9}$/L)	13.2	4-11	Mono ($\times 10^{9}$/L)	0.59	0.2-0.8
Neut ($\times 10^{9}$/L)	5.5	2-7.5	Basophil ($\times 10^{9}$/L)	0.03	0.01– 0.1
PLT ($\times 10^{9}$/L)	282	150-400	Eosinophil ($\times 10^{9}$/L)	5.6	0.04 -0.4

Q1. What do you think the underlying diagnosis may be?

Q2. How confident are you that this will be the correct diagnosis?

Not confident | Moderately confident | Very confident

02:34

[redacted]

Ryan

Vital Signs

RR: 23 breaths/min

Temp: 38.1 °C

BP: 140/80 mmHg

HR: 80 bpm

O2 sats: 95% -room air

Next Case

Case 3

03:21

[redacted]

Mohammad

Mohammad is a 25-year-old fitness instructor who presents to your surgery one evening with some vague flank pain after a heavy workout. He is usually fit and well, doesn't drink or smoke and is adopted.
While examining Mohammads's abdomen, you are surprised that you are able to feel his left kidney. You wonder if this is perhaps slightly bulky and you order a renal ultrasound.

The following week a very worried Mohammad returns after his scan, and as you review the results, to your dismay you see that he has some cysts in his kidneys. The radiologists report suggests this may represent Autosomal Dominant Polycystic Kidney Disease(ADPKD).

You call your local nephrologist, who tells you that in a man of Mohammad's age, an ultrasound scan is 95% sensitive in detecting ADPKD and has a 5% false positive rate. He tells you, in your local area, ADPKD has a prevalence of 1 in 1000 and is autosomal dominant in its inheritance.

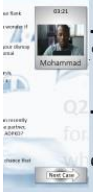

[illegible]. What is the approximate chance that Mohammad has ADPKD?

[illegible]% ☐ 2% ☐ 5% ☐ 9.5% ☐ 95% ☐

Q2. Mohammad is worried about this future children. He already has one child who is well and has a scan recently for an unrelated reason – This child certainly doesn't have ADPKD. He wants another child with the same partner, who does not have ADPKD. If Mohammad does have ADPKD, what is his second child's chance of having ADPKD?

Greater than the first child ☐ Less than the first child ☐ The same ☐

Q3. If Mohammad has a child with a different partner, who is confirmed not to have ADPKD, what is the chance that this child will have ADPKD.

Greater than the first child ☐ Less than the first child ☐ The same ☐

Next Case

Case 4

04:11

[redacted]

James

A disgruntled ED nurse hands you a patient file. "James Smith is back again" she grumbles.

James is well known to the department, and the nursing staff. He attends on a few occasions per month in exquisite pain, the source of which is never found, but will only respond to opiates. James has a colourful history and is currently an active intravenous drug user and has attended with at least 1 heroin overdose and related long lie in the last 6 months.

James is hunched over in the bed when you arrive. He tells you he has 11/10 pain in his abdomen and shooting down his groin into his leg. It has been worsening over the last day, and he feels absolutely terrible.
He knows that nothing is going to relieve this pain except for that drug that begins with the "O", but he can't remember exactly as he never pays much attention to drug names.

"Oxycodone?" volunteers the nurse.
"Yeah, that's the one alright! Nothing else helps!"

He won't let you lie him down full for an abdominal examination and swipes your hand away when you attempt to place it on his abdomen.
"11/10, didn't you hear me? What more do you need to know?"

Vital Signs

RR: 24 breaths/min

Temp: 38 °C

BP: 120/70 mmHg

HR: 110 bpm

O2 sats: 95% -room air

Q1. What is your clinical impression of this situation? ☐

Q2. Based on your answer to Q1, what analgesia would you offer James at this point? ☐

Q3. What investigations , if any, would you perform next? ☐

Next Case

Case 5

05:14

Paul

Paul is a 19-year-old rugby player whose mother drives him to your emergency department as he has been unwell for 24 hours. She tells you Paul played a rugby match yesterday, and was feeling a bit unwell before the game, with some flu like symptoms. His mother jokes that he really did not play well at all, and even had to get taken off early after he received some very heavy tackles.

Paul has been getting worse since his game, took to bed with his aches and pains and now with an additional headache and slept all evening - in fact has been in bed since the match. When his mother roused him this morning, he complained of some positional feeling unwell and having some chest pain so she gave him an over the counter painkiller from her drug cabinet, she isn't sure which one.

His mother is worried about his chest pain, as there is a strong history in her family and Paul's father had a serious heart attack at age 42.

Paul looks reasonably well to you, although he is dosing off in the chair as he sits next to his mom – she really can't remember what that painkiller was, but is very sleepy!
Paul's blood results return and show you that his admission trop is at the highest normal value on your hospitals reference range. To your eyes, his ECG certainly has some ST changes beginning diffusely.

Q1. Is it likely this boy is having a heart attack as his mother suggests?

Q2. List 2 potential diagnoses

Q3. What actions would you take next.

Vital Signs

RR: 15 breaths/min

Temp: 37.1 °C

BP: 120/70 mmHg

HR: 65 bpm

O2 sats: 95% -room air

Next Case

Case 5

05:14

Paul

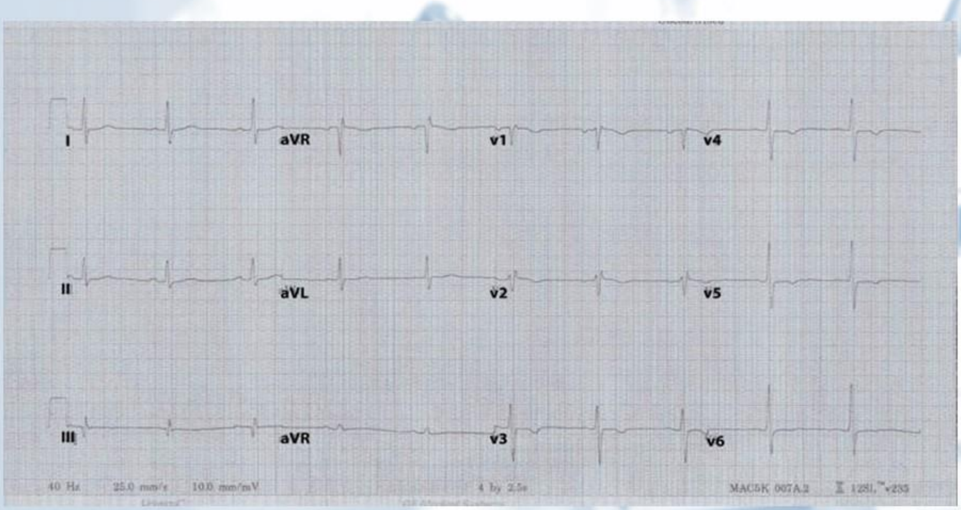

Vital Signs

RR: 15 breaths/min

Temp: 37.1 °C

BP: 120/70 mmHg

HR: 65 bpm

O2 sats: 95% -room air

Return

Case 6

06:34

Margaret

Margaret is a 30-year-old female who has been referred to your clinic by her GP with a non-productive cough, malaise and mild chest discomfort.

She is a mother of 2 young children who are unwell at home and she has been struggling to take care of them these last few days due to her own illness. She was seen by the medical consultant on duty, who noted her respiratory rate increased, clear chest Xray and her D-dimers were slightly raised. She has a background of CKD 3 from reflux nephropathy; her baseline eGFR is 30 mL/min per 1.73 m^2.

The consultant is concerned that Margaret has a pulmonary embolism, and commences her on heparin in advance of any imaging as it is late in the evening and they expect some delays before any scan can be performed.

The next day you receive a call from radiology asking you if you want to perform a CT pulmonary angiogram, with the contrast load and risk to her kidneys which that entails, or a V/Q scan, which is a more difficult scan to interpret and may miss a PE.

Margaret tells you she is worried about her kidneys, but also worried that she may have a clot in her lungs as she knows of people who have died from this condition.

Vital Signs

RR: 23 breaths/min

Temp: 38 °C

BP: 120/70 mmHg

HR: 87 bpm

O2 sats: 95% -room air

Question: How do you proceed next?

Answer: 150 words max

Next Case

Case 7

07:55

Lisa

Lisa was admitted with fever, consolidation on his chest X-ray and raised white blood cell count. A diagnosis of pneumonia was made.

You reach for your local antibiotic protocol and see that you have two options to choose from, antibiotic A and antibiotic B. You estimate that Lisa will need 3 doses of antibiotic daily for a week. The protocol has a brief summary of each as follows:

Antibiotic A was poorly tolerated with a number of side effects, most worryingly causing irrecoverable bone marrow suppression and in clinical trials of patients with pneumonia resulted in 1 of every 50 patients taking the drug dying. It is **substantially** cheaper however then any alternatives.

Antibiotic B is a newer enhanced class of drug which had only minimal side effects, limited only to mild GI disturbance and in randomised control trials in patients with pneumonia was shown to lead to 95% survival. In contrast however, it is a relatively expensive drug, at 300 pounds a dose.

Q1. Assuming this is a public health care system such as the NHS, which drug would you give to this patient?

Q3. Would your approach change if this patient were in a private hospital and had to pay the cost himself?

Q4. If so, how would your approach differ? 150 words max

Next Case

Case 8

08:59

Alice

Alice attends your clinic unwell for two days. You listen to her symptoms and you think she is suffering from a common cold. Alice is a medical student, and is convinced something more serious is going on – she has been reading too many textbooks lately. You try to reassure Alice and help her identify in herself the symptoms of the common cold.

Question

Based on the classic symptoms that you would expect to see in a common cold, rank the following symptoms in order of likely hood of being present in Alice (1 = most likely, 5= least likely)

(a) Runny nose and headache ☐
(b) Fatigue ☐
(c) Ear pain and shortness of breath ☐
(d) Sore throat ☐
(e) Headache ☐

Next Case

Case 9

09:54

Meg

Meg is an 18-year-old female who has just been referred by her General practioner to a psychiatric service for symptoms of severe anxiety and depression. She has been having frequent episodic dyspnoea, associated with hyperventilation, carpopedal spasm, and loss of consciousness. The admitting psychiatrist wants to exclude the possibility of a respiratory infection and sends the patient to the emergency department (ED) with a referral letter asking to out rule a pneumonia.

You meet Meg and note she is not in significant distress other than feeling quite breathless. She is obese, has a history of asthma, and smokes 15 cigarettes per day.

She is currently being treated with a benzodiazepine and anxiolytics and is taking a birth-control pill. Her chest and cardiovascular examination are normal.

Her chest X-ray is officially reported as normal by the radiology department, and her routine blood test are normal. You reassure Meg that all is well and she can return to the psychiatric ward. She clearly isn't particularly pleased about going back to the psychiatric unit, but gives nods and gives you a short smile and stares out the window as you leave.

While waiting for the ambulance transfer, Meg becomes very agitated and short of breath as the nursing staff repeat her observations.

Vital Signs

RR: 35 breaths/min

Temp: 37.2 °C

BP: 105/60 mmHg

HR: 110 bpm

O2 sats: 93% ~room air

Q1. What is your diagnosis and what is the first line therapy? ☐

Q2. Are any further tests necessary here? ☐

Next Case

Case 10

Peter is a 19-year-old rugby player, who presents feeling unwell after playing a particularly intense match yesterday. You realise he is a team mate of one of the patients you saw earlier in your shift, Paul.

Peter has been feeling unwell these last few days, and has taken to bed with his aches and pains. When he awoke this morning after yesterdays match, he complained of some mild positional chest pain and he took a few ibuprofen tablets and felt quite a bit better.

Peter looks reasonably well to you and as your cardiovascular examination is completely unremarkable. His vital signs are all normal. He does have some pain on deep breathing and it is certainly worse in certain positions.

Peters blood results return and show you that his admission trop is at the highest normal value on your hospitals reference range. To your eyes, his ECG is probably normal, although you wonder about a little high take off.

Q1. What is the likely diagnosis

Q2. What tests, if any would you request next

Q3. What is your treatment plan

11:14

Peter

Vital Signs

RR: 16 breaths/min

Temp: 37.2 °C

BP: 120/70 mmHg

HR: 87 bpm

O2 sats: 99% -room air

Finish
