# Supplementary material for: A cognitive forcing tool to mitigate cognitive bias – a randomised control trial
Source: BMC Med Educ. 2019 Jan 8;19:12. doi: 10.1186/s12909-018-1444-3 (PMC6325867; doi:10.1186/s12909-018-1444-3)
Supplement: Supplementary file 3 — Qualitative interview summaries. More complete summaries of the themes emerging form the qualitative interviews. (DOCX 23 kb) [file 12909_2018_1444_MOESM3_ESM.docx]

Appendix 3

### The cases in detail

### Case 1: Deliberate red herrings suggesting HIV

Bias: Representativeness bias

*“well this one is fairly obvious, he has had risky sex”*

- Doctor immediately latches on to the less likely representation of the patient and does not consider alternatives

Representativeness bias here is the degree to which Gary is similar in essential characteristics to his parent population. Flu is statistically far more common in this patient, and his parent population is simply healthcare workers. Increasing his flu risk, he has skipped his vaccinations and isn’t looking after himself. The idea of HIV is planted by mentioning Gary has had unprotect sex with a man. Representativeness bias causes the candidate to identify Gary instead as a member of a much rarer population (HIV+ men) simply because he has nonspecific features of that much less likely population, i.e. he belongs more to the unvaccinated healthcare group than high risk HIV+ men, but this is incorrectly deduced.

Most participants felt that HIV was the most likely diagnosis. Interestingly, as clinicians became more senior they increasingly conceded that viral infection was an important differential and often the most likely, but many still felt more concerned about HIV. Often participants considered flu, but once they had identified the risk factors for HIV, they rarely reconsidered any other diagnosis and became fixated on this.

### Case 2: This is a tricky diagnosis, based on a real case of Churg-Strauss syndrome.

Bias: Overconfidence bias.

“Something is going on, but I’m really not sure what. I know I’m missing something”

- Lack of confidence here mirrored uncertainty, this was a correct response

Overconfidence Bias occurs when a candidate’s confidence is high despite incorrect answer. Most participants were uncertain of the diagnosis here and this is reflected in the wide variety of differentials offered. None were confident enough as they worked through the case to commit firmly to a definite diagnosis, thus there was little evidence of over-confidence bias based on these conversations.

### Case 3: Prevalence of polycystic kidney disease

Bias: Unawareness of statistics and probabilities, base rate neglect.

All participants struggled to conceptualise the true incidence of ADPKD here, and failed to consider the local prevalence, which is of course almost always the correct population to consider. The crux of this case is the high false positive rate. Almost all confused sensitivity with post-test probability, overall giving the impression that understanding of likelihoods and diagnostic tests was poor, and base rate neglect was common. All candidates groaned and where unhappy with this question, and almost immediately identified this as something they were confused by, showing a degree of metacognition and insight.

The correct answer is 2%. This is a rare disease with a prevalence of only 1 in 1000. 95% sensitivity and a 5% false positive rate means that every scan that comes back positive is true 95% of the time, and falsely positive 5% of the time.

Imagine a scenario 100,000 people are tested for ADPKD. Since this effects 1 in 1000 people, we would expect 100 people to have the disease. Of this people, given the 95% sensitivity, 95 /100 will be correctly identified as having ADPKD.

However, this was from a screening of 100,000 people. That means that 99,900 people would not have the disease. Of those 99,900, 4,995 (i.e. 5%) people would receive a false positive diagnosis. That compares to only 95 people receiving a correct positive diagnosis. Therefore, the probability upon receiving a positive test that one has the disease is the number of true positives divided by the number of false positives = 1.9% (95/4,995)

The key fact that makes it very unlikely that Bill has the disease is the fact this is a rare condition, and this was in effect a screening test – i.e. the BASE RATE is low, and as such, and false positive in a test will have significant consequences.

### Case 4: Potential Drug seeking behaviour (underlying serious infection)

Bias: Confirmation bias

*“I know he wants opiates, and I’m not a cruel doctor, so I don’t want to judge him and withhold proper analgesia in this gentleman”*

-fixating on the ethical issues immediately thus consolidating the confirmation bias

This is a classic “boy who cried wolf” scenario. The scene is created of a regular hospital attender exhibiting drug seeking behaviour. The bias here, is that the scene “confirms” the candidate’s prior suspicion of drug seeking behaviour and no further effort is made to appraise the situation. However, this is not a normal scenario – James is tachycardic and has a fever. In an IVDU, this is a worrying sign and raises questions of infection – endocarditis, abscess etc. The pain shooting down his leg hints at the latter.

The red herring here was quite effective: trying to frame this as an ethical dilemma pertaining to judging drug seekers and giving them analgesia. This further leads the reader towards a confirmation bias. Most candidates immediately began to reflect on the difficulties of administering opiates, and the moralistic nature of judging people like this. The broader questions were distracting them from the patient. There was an apparent change in attitude amongst candidates as their seniority increased. The more junior doctors and students immediately gravitated to the drug seeking phenotype and ignored the warning signs and red flags, whereas more senior doctors were open to the fact that drug seekers can still suffer from serious illness, and the registrars (post grad year 4+) almost universally were concerned about infection. This suggests perhaps that some biases will reduce with time and experience.

### Case 5: A concussed rugby player with a distracting mother

Bias: Suttons slip a.k.a “when you hear hoofbeats: think horses, not zebras”, search satisfying

*“I think we can be quite confident in reassuring this woman, he is too young for an MI”*

- Focusing on mother and the rare diagnosis, rather than the patient

The bias here is a variation on “Suttons slip” which occurs when the candidate correctly advises the mother that this boy is not having a heart attack (she is worried about zebras) and is distracted from the true diagnosis by her anxiety and questions. However, the true diagnosis here is of concussion or intracranial event, based on the history of somnolence, headache and more unusual ECG changes. This is a more difficult diagnosis to put together.

A common theme here was being distracted by the mother and focussing on mother’s concerns of MI, and candidates allowing themselves to be swept along by the conversation. Once they had sufficiently satisfied themselves that this boy was not having a heart attack, many candidates stopped thinking about the case, or continued to ruminate on cardiac disease (wondering about pericarditis was a common finding – perhaps an availability bias). Only a few of the more senior candidates realised the correct issue here – each time this happened, they had essentially stopped and “taken a step back” to assess the entire situation in its entirety.

“hang on a minute, stop. This guy just took some big tackles and now is drowsy in front of me… get him into a scanner!”

A second issue was the people were commonly confused by “highest normal range troponin”, disregarding the fact that it was still normal, and thinking that it must have some relevance because I mentioned it. This may have been a limitation of my own question writing and clarity rather than any true confusion on the part of the candidates. Overall, the tone of the entire case, and the doctors thought process was powerfully influenced by the anxious mother.

### Case 6: Consultant incorrectly suspects a PE

Bias: Diagnostic momentum

“I think we need to take a step back here and reassess, this doesn’t strike me as a PE”

- Resistance to the diagnostic momentum

Margaret’s story is inconsistent with a PE, as are her vital signs. D-Dimers can be raised in both CKD and simple infections, which is more consistent with a person caring for multiple virally unwell children.

In general, candidates were quite resistant to diagnostic momentum here, perhaps because there was a clear power structure that they were aware of an aware of the dangers of believing in. One candidate mentioned that it was easy to go against an imaginary consultant, which highlighted for me the limitations of such interviews and how closing they might reflect real life behaviours. As compared to the previous case, many candidates correctly felt that they should stop the current diagnostic pathway and they correctly revaluated the information provided. Senior doctors were more likely to do so than juniors/medical students. This is an interesting contrast to the quantitative data where very few were resistant to diagnostic momentum.

### Case 7: Choosing between two antibiotics

**Bias: The Framing effect**

“This one is easy, antibiotic B. Bone marrow failure and death are bad”

- Being driven away from the superior choice because of highlighting the negative side effects.

This question uses the price as a red herring to fool the candidates into focusing on drug costs and ethics. However, the key fact is that antibiotic A is more effective (2.5% of patients die versus 5% with antibiotic B) and cheaper, and is unequivocally the correct answer. Choosing antibiotic B reflects the student being influenced by the framing effect, where the “positive spin” on the efficacy of antibiotic B masks the fact it is inferior in every way.

About half of the candidates chose the incorrect drug here, succumbing to the framing effect. Once one can overcome the framing effect, objectively, the information presented was very clear with no ambiguity. Listening to people reacting with negative emotions (dismay, caution, fear) to the words death/marrow failure/price, and not look at the objective data was disheartening. This will not be news to advertising agencies or drug company executives, but I had hoped doctors were more aware of this phenomenon.

### Case 8: A medical student with flu

Bias: Conjunction bias occurs if (a) is ranked more highly than (f)

The majority of candidates incorrectly chose headache and running nose as being more common than headache. As candidates discussed and reflected on this, I worried that there was a misunderstanding on their part, and quite a number of them felt that headache equated to “just headache”. In their defence, it is entirely possible that it is more common to have a headache and runny nose together than it is to have a “just a headache” in viral illness. The question was rather an attempt to probe candidates’ understanding of the fact that every patient with symptoms A and B, must by definition have symptom A, therefore it is truer to say that symptom A, in all its forms, has a greater prevalence than A and B in combination.

On reflection, I think this question is not a good test of this concept and the results are not meaningful.

### Case 9: Psychiatric patient with a Pulmonary Embolism(PE)

Bias: Framing Effect, Momentum.

“This doesn’t quite add up for me. Anxiety shouldn’t make you hypoxic”

- Attention to the objective measurements help many candidates realise there was more to this case than initially suspected.

This person has a PE, with multiple risk factors. Framing and momentum will lead to incorrect diagnosis of anxiety syndrome.

This was a very interesting case based on a real-life example where most people succumbed to the well-documented institutional bias against people with psychiatric illness. So powerful was this effect, that a few candidates noticed the low oxygen saturations and tachycardia, and even commented that this was unusual in anxiety, and yet continued to make the diagnosis of “anxiety”. As in the real case, most candidates immediately viewed the case through the lens of psychiatric illness, and didn’t correctly identify the risk factors for a PE and note that the objective vital sign measurements were a concern.

### Case 10: Chest pain post rugby

Bias: Availability bias

“So, this is another chest pain. This one isn’t cardiac either”

- Correct diagnosis, but acknowledging the earlier case, and immediately refuting a cardiac diagnosis, although it’s not explicitly requested.

The correct answer here is muscular skeletal pain, or flu like symptoms. Case 5 was deliberately very similar to case 10 (purposely designed this way to trigger the memory of case 5 in candidates) which had hinted at cardiac disease. Availability bias would occur when the respondent suggested a cardiac diagnosis more often because of this previous exposure.

Availability bias is hard to assess, as it is not clear if an incorrect answer is truly because of availability bias, or simply just an incorrect assessment of the situation “de novo.”

Because of this, half of the questionnaires had swapped case 5 and case 10. If there is a similar number or respondents suggesting cardiac disease in both, then it is likely that this question is not triggering an availability bias. However, 7/10 candidates exposed to case 10 after prior “priming” with case 5 strongly entertained cardiac disease in their differential, as compared to 3/10 in the group exposed to case 10 first. This implies such a format might well trigger at least a degree of availability bias.

Most candidates note the similarly between cases and immediately start contrasting the two cases, at least confirming that the similarly is noted.

Interestingly, many candidates now appreciated that musculoskeletal pain is very common after a game of rugby, and very few considered cardiac causes at any great length. The lack of a concern mother voicing specific diagnosis was enough to allow them to step back and be more objective. Some reflected that they may have given incorrect responses to case 5 now that they were thinking about this more objectively with an anxious mother in the room.
